# “*Early in the morning, there’s tolerance and later in the day it disappears*” – The intersection of resource scarcity, stress and stigma in mental health and substance use care in South Africa

**DOI:** 10.1017/gmh.2024.41

**Published:** 2024-04-01

**Authors:** Abigail C. Hines, Alexandra L. Rose, Kristen S. Regenauer, Imani Brown, Kim Johnson, Jessica Bonumwezi, Sibabalwe Ndamase, Nonceba Ciya, Jessica F. Magidson, Bronwyn Myers

**Affiliations:** 1 Department of Psychology, University of Maryland, College Park, MD, USA; 2 Mental Health, Alcohol, Substance Use, Tobacco Research Unit, South African Medical Research Council, Parow, South Africa; 3 Center for Substance Use, Addiction & Health Research (CESAR), University of Maryland, College Park, MD, USA; 4 Curtin enAble Institute, Faculty of Health Sciences, Curtin University, Perth, Western Australia, Australia; 5 Department of Psychiatry and Mental Health, University of Cape Town, Cape Town, South Africa

**Keywords:** stress, stigmatization, addiction, global mental health, quality of care, non-specialist provider

## Abstract

Stress is a challenge among non-specialist health workers worldwide, particularly in low-resource settings. Understanding and targeting stress is critical for supporting non-specialists and their patients, as stress negatively affects patient care. Further, stigma toward mental health and substance use conditions also impacts patient care. However, there is little information on the intersection of these factors. This sub-analysis aims to explore how substance use and mental health stigma intersect with provider stress and resource constraints to influence the care of people with HIV/TB. We conducted semi-structured interviews (*N*=30) with patients (*n*=15) and providers (*n*=15, non-specialist health workers) within a low-resource community in Cape Town, South Africa. Data were analyzed using thematic analysis. Three key themes were identified: (1) resource constraints negatively affect patient care and contribute to non-specialist stress; (2) in the context of stress, non-specialists are hesitant to work with patients with mental health or substance use concerns, who they view as more demanding and (3) stress contributes to provider stigma, which negatively impacts patient care. Findings highlight the need for multilevel interventions targeting both provider stress and stigma toward people with mental health and substance use concerns, especially within the context of non-specialist-delivered mental health services in low-resource settings.

## Impact statement

Non-specialist health worker stress is common in low-resource settings and impacts both healthcare workers and the patients they serve. Stigma toward mental health or substance use is also a well-documented issue among healthcare workers. This study explores the relationship between non-specialist provider stress and stigma toward mental health and substance use problems, and how these may intersect to diminish the quality of patient care. It highlights the need for multilevel interventions targeting both provider stress and stigma to best support non-specialists to provide effective substance use and mental health services. This study has implications for task-shared mental health training programs and highlights the importance of including provider stress and stigma as training targets alongside clinical knowledge and skills development to ensure training leads to high-quality patient care. This study also helps to represent the perspectives of patients receiving task-shared mental health services within the global mental health literature.

## Introduction

Globally, there is increasing awareness of stress and burnout, a syndrome resulting from chronic work-related stress among healthcare workers (De Hert, 2020; Moss and Good, 2016; World Health Organization & Burton, 2010). Stress is a particular challenge in low-resource health settings, where healthcare workers face high caseloads and are expected to treat complex cases in chronically understaffed and overcrowded environments (Dugani et al., 2018; Wright et al., 2022). To ameliorate shortages of specialist providers, some health care teams have expanded the use of non-specialist health workers, including community health workers (CHWs) and nurses (Kigozi et al., 2020; Owuor et al., 2020; Padmanabhanunni, 2020; Jensen et al., 2022). In South Africa, non-specialists were originally deployed to provide and increase retention in HIV/TB care (Simelela and Venter, 2014). However, as South Africa has made significant progress in treating HIV/TB, the roles and responsibilities of non-specialists have expanded to include other health priorities, leaving these workers particularly vulnerable to stress and burnout. For instance, a recent systematic review highlighted high levels of burnout among nurses working across a range of settings in sub-Saharan Africa who were not specially trained for their responsibilities (Owuor et al., 2020). Similarly, a study in the Western Cape estimated that about half of CHWs working in nongovernmental organizations indicated burnout and secondary traumatic stress (Padmanabhanunni, 2020).

In South Africa, people with mental health (MH) and substance use (SU) concerns are overrepresented in HIV and/or TB care (Janse Van Rensburg et al., 2020; Myers et al., 2021; Peprah et al., 2022). In sub-Saharan Africa, approximately 15.3% of people with HIV (PWH) have comorbid major depression (Lofgren et al., 2020). In a South African sample of patients with HIV, approximately 37% indicated harmful drinking and 13% indicated drug use (Kader et al., 2014). Yet these patients experience limited access to mental healthcare in the public sector. Crude estimates show that of uninsured South Africans who require mental healthcare in the public sector, between 1 and 7% actually receive care (Docrat et al., 2019). This is due to limited investment in MH services and shortages of MH specialists. South Africa’s expenditures on public MH are only about 5% of the total public health budget (Docrat et al., 2019), and as of 2019, there were only 0.38 psychiatrists per 100,000 people in the public sector (Wishnia et al., 2019). Consequently, non-specialists are being increasingly relied upon to address gaps in the public MH system, referred to as “task sharing.” This involves the shifting of MH counseling from specialist providers to non-specialists (Department of Health Republic of South Africa, 2013; Sorsdahl et al., 2023). However, non-specialists get little training in MH/SU concerns and may be poorly equipped to actually take on additional roles (Schneider et al., 2016). Providing clinical services to patients that non-specialists have limited exposure to or knowledge of, such as patients with MH or SU, may exacerbate provider stress (Makhado and Davhana-Maselesele, 2016; Regenauer et al., in Press). While the relationship between MH literacy and stress in low- and middle-income countries is in its nascence, there is some evidence that increasing MH literacy helps reduce provider stress (Agyapong et al., 2023).

Non-specialists in HIV/TB care also face systemic frustrations when providing MH or SU care, which likely impact stress. For instance, HIV care providers in South Africa reported that referral processes to formal MH/SU services were unclear or time-consuming, even when such referrals were to a co-located clinic (Sorsdahl et al., 2021; Belus et al., 2022). Additionally, when task sharing of MH/SU services is implemented without providing additional support or appropriate compensation to non-specialists, this can be perceived as “task dumping,” increasing their work-related stress (Jacobs et al., 2021; Sorsdahl et al., 2021).

Addressing non-specialist stress is critical not only to support their well-being but also of patients, as provider stress is associated with more stigmatizing behaviors, or enacted stigma, toward patients (Kim et al., 2018; Eshun-Wilson et al., 2019; Tawfik et al., 2019; Román-Sánchez et al., 2022). Stigma is multidimensional and has many facets, such as enacted stigma (when others treat stigmatized groups with prejudice or discrimination), internalized stigma (when stigmatized individuals view themselves with lower value or worth) or anticipated stigma (expectations from stigmatized individuals that others will discriminate against them), making it complex to study (Pescosolido and Martin, 2015). This conceptual complexity makes it important to distinguish what types of stigma are relevant to a given situation (Griffith and Kohrt, 2016). Research among adults living with HIV in Ethiopia found that high workload among staff led to a lack of empathy and rushed work, as reported by patients, which deterred patients from accessing care, possibly demonstrating enacted and internalized stigma (Bezabhe et al., 2014). Further, stigma from non-specialists toward PWH and TB with MH or SU problems also exists independently of provider stress levels (van Boekel et al., 2013; Regenauer et al., 2020). Several studies in South Africa have found that non-specialists are reluctant to screen for and intervene with MH or SU problems due to stigmatizing beliefs (van Boekel et al., 2013; Myers et al., 2019). For instance, a qualitative study identified non-specialist stigma toward people with MH concerns as a barrier to nonspecialists taking on MH care duties in integrated primary care settings (Mendenhall et al., 2014). In our prior work with non-specialists (*n*=17), which included CHWs and nurse supervisors, the average baseline Social Distance Scale scores (possible range 6–24) toward patients with depression or SU were 7 and 14, respectively (Regenauer et al., in Press). These findings indicate the likelihood of higher levels of stigma toward patients with SU, which may be a particular barrier for these patients.

Therefore, while stress and stigma may independently impact the quality of care for patients with MH/SU conditions, they are also likely interrelated. Yet, little is known about how non-specialist stress, stigma and resource challenges affect their care for PWH and/or TB in South Africa. Being aware of the challenges faced by non-specialists in delivering services for patients with MH/SU concerns, including patient perspectives, could inform task-sharing programs going forward. This paper aims to qualitatively explore how stress and stigma interplay to influence non-specialist-delivered MH/SU care for PWH and/or TB in the South African healthcare system, from both patient and non-specialist provider perspectives.

## Methods

Semi-structured, individual interviews (*N* = 30) were conducted between February and June 2021 with patients (*n* = 15) and non-specialist health workers (*n =* 15) from HIV/TB and SU treatment clinics in low-income areas with high HIV/TB and SU burdens in Cape Town, South Africa. The primary aim of the parent study was to inform the development of an intervention to help reduce MH/SU stigma among providers and improve engagement in HIV care for PWH with co-occurring MH or SU problems (Myers et al., 2024). The interview guide focused on stigma (primarily enacted stigma) and strategies for stigma reduction among non-specialists (Rose et al., 2023; Regenauer et al., in Press). Enacted stigma can be defined as “negative attitudes expressed by members of the public that are experienced by an individual with devalued characteristics” (Molina et al., 2013). Purposive sampling (Palinkas et al., 2015) was used to recruit participants who could provide insight relevant to the study aims. Our sample size (*N =* 30) was expected to meet the criteria for theoretical saturation (Hennink and Kaiser, 2022), and coders identified that saturation had been reached at this point as there were no major themes that were newly emerging once all transcripts had been reviewed and analyzed. The parent study aims were to explore patient and non-specialist provider perspectives on their experiences and attitudes on working with patients with MH/SU concerns at HIV/TB clinics. Unprompted, participants also spoke about resource limitations and stress as it related to caring for these patients, which led to the current sub-analysis.

### Participants

The Western Cape Provincial Department of Health, which oversees health services in the Western Cape Province, assisted in identifying non-specialists in HIV care to approach for this study. The HIV care cascade can be described as screening and diagnosis, linkage to care, retention in care, initiation of antiretroviral therapy (ART), retention on ART, and viral suppression (Mugglin et al., 2021). Different providers play key roles in select areas of this cascade, with nurses involved in diagnosis, linkage to care and initiation on ART, whereas CHWs are involved in the parts of the cascade surrounding adherence counseling, support, and retention in care. The scope of non-specialist interaction with patients varies, ranging from direct interaction within the clinic (nurses), home visits (CHWs) and supervisory roles (program managers). Non-specialists were included if they were: (a) directly involved in community-based HIV care services; (b) a facility-based HIV provider interacting with community-based teams; (c) managed community-based HIV services or (d) involved in SU treatment. All non-specialists worked for the state or in nonprofit organizations receiving public funding for HIV care delivery, were >18 years old, and were able to provide informed consent and complete interviews in isiXhosa or English, the main languages spoken in the target communities.

Referrals from non-specialist provider participants were used to purposively identify patients. Patients were included if they self-reported: (1) being >18 years old; (2) living with HIV; (3) struggling with HIV care engagement (e.g., missed appointments); (4) active SU (i.e., >2 on the AUDIT-C; (Bush et al., 1998; Morojele et al., 2017) or using >1 illicit drug in the past 3 months) or suspected depressive symptoms (>2 on the PHQ-2 (Gilbody et al., 2007); adapted from the PHQ-9, which had been previously validated in South Africa (Bhana et al., 2015)); and were (5) able to complete informed consent and interviews in isiXhosa or English. Table 1 provides an overview of the participant demographics.

**Table 1.** Demographics of the sample

Variable	Total *(N =* 30)	Non-specialists (*n* = 15)	Patients (*n* = 15)
	*M (SD)*	*M (SD)*	*M (SD)*
Age, *M (SD)*	41.5 (10.61)	45.5 (8.6)	37.5 (11.1)
Race, % *(n)*	83.3 (25)	86.7 (13)	80.0 (12)
Identified as female, % *(n)*	76.7 (23)	86.7 (13)	66.7 (10)
Education (completed high school or above), % *(n)*	53.3 (16)	73.3 (11)	33.3 (5)
Prior MH/SU training, % *(n)*	—	66.7 (10)	—
Time in position, % *(n)* 0–6 months	—	20.0 (3)	—
6–11 months	—	20.0 (3)	—
1–3 years	—	20.0 (3)	—
3–5 years	—	6.7 (1)	—
Over 5 years	—	33.3 (5)	—
MH or SU–related problems	—	—	100 (15)

### Procedures

Interviews were conducted by trained research assistants (RAs) in isiXhosa or English based on participant preference. Prior to the interview, the RAs obtained written informed consent and collected demographic information from the participants. Using a semi-structured interview guide, RAs asked participants about their experiences of stigma around HIV, TB, SU and MH, as well as specific feedback to inform the development of a MH/SU stigma-reduction intervention for providers. Patient and stakeholder interview guides were developed separately in both isiXhosa and English. The Link and Phelan stigma framework, the Situated Information Motivation Behavioral Skills Model of Care Initiation and Maintenance, and the Consolidated Framework for Implementation Research informed the development of the interview guide (Link and Phelan, 2001; Damschroder et al., 2009; Rivet Amico, 2011). The interview guides for patients and providers have been included as supplementary material.

Interviews were audio-recorded and had an average duration of 45 minutes. Participants were compensated 150ZAR (about $10 USD) in grocery vouchers upon interview completion. Audio-recordings conducted in isiXhosa were translated into English, and all interviews were transcribed verbatim using Otter.ai (Corrente and Bourgeault, 2022). Transcripts were double checked for quality assurance and were imported to NVivo (2020) for coding.

All procedures were approved by the Human Research Ethics Committee at the South African Medical Research Council (EC039-9/2020) with an Institutional Review Board Authorization Agreement with the University of Maryland.

### Data analysis

We used a hybrid inductive–deductive approach for codebook development and analysis. The parent study had specific aims to use interview coding to guide the development of a stigma reduction intervention and thus our approach was partially deductive. However, during interviews and upon transcript review, it became apparent that participants were also raising other themes, such as those focused on provider stress, so we also took a partially inductive approach to codebook development (Fereday and Muir-Cochrane, 2006; Kiger and Varpio, 2020). Given the focus of the parent study, there was less direct probing on resource scarcity and stress, though both topics were organically brought up by most participants and featured prominently throughout the interviews. An initial codebook was developed by using the interview guide and by open coding of several transcripts. Specifically, South African study team members with deep knowledge of the study context and US-based team members held group discussions of several open-coded transcripts to identify any additional inductive codes not already included in the interview guide. These codes were then added to create the final codebook, which was then piloted and presented back to the larger team for further refinement before coding. Transcripts were coded by two independent coders who held weekly meetings to review and resolve coding discrepancies. Local South African team members were available throughout the coding process to provide input on interpretation and to discuss main themes. The inter-rater reliability (IRR) among coders was 0.88. This was above the IRR of 0.80 set prior to the coding process, which is considered adequate as supported by the literature (O’Connor and Joffe, 2020).

## Results

The sample was evenly distributed between patients (*n=*15) and non-specialist providers (*n=*15). Non-specialist providers included nurses, CHWs, counselors and program managers. The average age of patients was 37.5 years (SD = 11.1), and most patients were Black African (*n=*12) and female (*n*=10). The average age of providers was 45.5 (*SD* = 8.6). Most providers were Black African (*n=*13) and female (*n*=13), reflecting the demographics of the broader non-specialist workforce in this setting. Most providers had a University/Technicon degree (*n=*9), one had a doctoral degree or equivalent, one had completed high school and four had not completed high school. There was a range of provider experience, with 40% of providers having worked at their current position for less than a year and about 33% of providers having worked at their current position for over five years.

We identified three main themes: (1) staff resource constraints drive stress and affect patient care, (2) in the context of limited time and stress, providers view patients with MH or SU concerns as more demanding and (3) stress contributes to stigma and stigma exists independent of stress. Below we describe each of these themes.

### Theme 1: Overall, staff resource constraints drive stress and affect patient care

Non-specialist providers mentioned understaffing as an issue. Within clinical interactions, some patients shared that they felt clinical information regarding HIV (e.g., HIV medication options, side effects) was not explained fully to them and that it was difficult to obtain answers to their questions, suggesting that staff may be too overloaded with other work to spend time providing detailed information to patients.

“*… Their explanation, it’s half – it’s not complete. All they say is when you start your treatment you will experience side effects, that’s all they say. And so it made me to become frustrated because I had questions and it was difficult for me to ask those questions because they were also not specific…*”

– Male, Patient, 39

Participants reported that staffing constraints were a particular issue for MH care because of the shortage of trained professionals available. Participants described how lengthy waits for specialist referrals adversely impacted patient retention.

*“And the psychiatrist there like maybe they had a lot of times also they had to join a queue. Maybe the plan might be seen after two months, and that will be late. Because they say, “Ok, we are giving you an appointment for your evaluation next year” and the clients tend to also give up hope on it also.”*

– Male, Registered Nurse Counselor, 31

Both patients and providers thought these staffing constraints contributed to stress and in turn affected clinical care.

*“Yes, the way they treated me, the way they do things… when you go there, you go there knowing that you will be treated this way and you also go there knowing that you may be helped, you may not be helped, you may be sworn at, you expect anything to happen. You go there with the body that is bullet proofed.”*

– Male, Patient, 39

*“So I think early in the morning, there’s tolerance [from the non-specialists towards patients] and later in the day it disappears, so it’s directly related to what people need to do. I think for me[…], it’s how much pressure is on the clinic?”*

– Male, Program Manager, 54

### Theme 2: In the context of limited time and stress, providers view care for patients with MH or SU concerns as more demanding

Non-specialist provider participants described that when clinic providers are already pressed for time, they experience patients who require more time and support, namely those with MH or SU concerns, as particularly challenging. With an already exhaustive workload and lack of support, providers may feel less inclined to go the extra mile for patients that need additional care and/or referrals, such as those with MH or SU problems.

*“But if you tell me: “[Name], you now need to put in a special program for mental health in your clinics”, Then I’d say: “Don’t I have enough work already? Are our people not already being overworked? You going to make me now depressed by ordering me to do that”.”*

– Male, Program Manager, 54

Another provider highlighted the difficulty of coordinating care with additional staff members for patients with MH or SU concerns.

*“You know…that these are the clients you have booked for the doctor. The HIV care clerk or Substance abuse [clerk] must take out the files on time, and looking up for a folder make people become irritated that “We don’t see your folder.” All of that takes time for the client to be seen and we don’t…we fail to plan and strategize…So it is important that we work together, Clerk, Therapist, Sister [nurse], CHW and all of us, we need to work together. I think that strategy could work too.”*

– Female, Counselor, 37

Some patients also believed that their cases were perceived as too time consuming or burdensome for staff, leading to provider impatience.

*“…they (CHWs) should instead be patient and they should try and find out the exact problem, where’s the root of the [SU/MH] problem”*

– Male, Patient, 39

In addition to increased logistical challenges with care, non-specialist providers reported that many staff were unfamiliar with MH/SU conditions, effective treatments and referral options, which led to hesitation to work with patients with MH or SU concerns. One provider described how this lack of knowledge led to the desire to refer patients with MH/SU concerns to other providers.

*“…Sometimes you see, it’s being clueless you see?*… *they do not know what to do with this person. Like someone would say, “I’m going to connect you with the Social worker” you see?”*

– Female, Counselor, 37

### Theme 3: Stress contributes to stigma and stigma exists independent of stress

Several non-specialist providers described experiences where they had seen clinic staff, frustrated by limited resources and unsure how to help patients, shouting at or making fun of patients with MH or SU concerns. Participants connected provider resistance to engaging in the extra effort potentially needed to care for patients with MH/SU problems to stigmatizing beliefs that these patients were intentionally difficult.

*“I am saying that it is very common because there is very little understanding to why they are doing what they are doing, there is always an assumption that they are troublemakers, without really looking into why they are in the situation that they are in.”*

– Female, Program Manager, 53

A CHW highlighted how patients often prefer to work with them instead of clinic nurses because of the way the nurses treat patients.

*“HIV and TB patients…they prefer that they come in, get attended to quickly get their medication… So you’d find in these cases that they would actually choose us, they prefer us to assist them in this case, than the nurses… And then now the nurses become very harsh towards them. And for us, with us we understand the situation that they are going through, we know the pain that they are going through, whatever struggles that they are going through.”*

– Female, Community Health Worker, 43

However, participants also described how stigma is influenced by factors other than work-related stress. A clinic program manager gave an example of how a coworker used stigmatizing language toward patients with MH concerns.

*“You’ll find out that someone will go to the clinic and this person is really mentally challenged, and you will hear a sister saying “Yhuu…this one didn’t treat the gonorrhea and that is why today it has come to his/her head, it’s making him/her go crazy”, I mean such things! “This one is not mentally ill, it’s those STI’s he/she did not treat”, I mean! So we cannot leave ourselves behind and say, “we are not stigmatizing these people”, we also – health care workers – we do really take part in spreading this stigma.”*

– Female, Program Manager, 40

One patient described negative feelings they experienced after harsh treatment from clinic staff after having missed appointments or adherence difficulties.

*“You come here and maybe you’ve skipped on your month and then the person [clinic staff] would start to judge you and swear at you and say this and that…you want to die.”*

– Male, Patient, 44

Specific to patients using substances, participants reported that providers may be fearful of patients due to stigmatizing beliefs that patients with SU problems were inherently dangerous and unpredictable.

*“…like, what if this person whose got substance issues, would like hurt me? [… ] and then if you have those thoughts and you act along those thoughts, the person will easily pick it up that you … don’t like what you’re doing and they will end up getting a leeway to hurt you. Because they don’t want you because you don’t want them.*

– Female, Program Manager, 47

One clinic program manager explicitly described the clinic setting as exhibiting “structural stigma”:

*“So I think in that sense, there is […] what we call it structural stigma, the structure don’t like people that’s slow or don’t like people that can’t look after themselves.”*

– Male, Program Manager, 54

Among participants, only non-specialist provider participants used the terminology “stigma” when discussing negative treatment of patients with MH/SU concerns. Instead, patients expressed discontent regarding providers talking about them to others, providers being “rude”, and feeling as though they are being “treated differently.” One patient described an instance when a non-specialist provider was making jokes about their TB and depression. However, the patient then said that they felt that the non-specialist provider “remained professional” and that these interactions did not bother them. This suggests that patients may be acclimated to this behavior or are justifying it so that it will be less hurtful to them.

## Discussion

In an HIV care setting in Cape Town, South Africa, both patient and non-specialist providers described the lack of human resources in public health care, which exacerbated work-related stress for non-specialists and contributed to their lack of time for higher-need patients. These participants also described non-specialist stigma toward MH/SU concerns and the ways in which stress influences stigma while acknowledging that stigma occurs independently of stress. Stress among non-specialists contributes to enacted stigma, however stigma may be preexisting prior to the onset of stress, as MH/SU are well known to be stigmatized across contexts, including those with very high resources (Wogen and Restrepo, 2020). This is consistent with the prior literature in South Africa, which states that the well-being of healthcare workers is an important aspect of delivering quality care programs, and that being overworked and stressed can lead to less effective communication from non-specialists to patients (Jensen et al., 2022). Findings suggest that non-specialist stress must be addressed alongside stigma to ensure providers are able to provide adequate patient care in the context of task-shared MH/SU services.

Notably, while provider wellness may typically be conceptualized as a system challenge and stigma as an individual-level outcome, both stress and stigma can be addressed at multiple levels (Rollins et al., 2021). Increasing funding and resource allocation for integration of task-shared MH/SU services in HIV/TB services may help overcome staffing constraints and stress among staff responsible for these services (Myers et al., 2022; Sorsdahl et al., 2023). Effective and thorough training of staff on MH/SU may make treatment of these patients feel more manageable to staff and thereby reduce stress and stigma. If staff are better trained, they may be more capable, feel more confident in their ability to provide quality care to all patients, and view patients differently (Sibeko et al., 2018; Jacobs et al., 2021; Agyapong et al., 2023). However, there is also evidence that changes in stress and stigma do not correlate (Román-Sánchez et al., 2022), and while additional training on new clinical topics can be perceived as helpful, it can also be perceived as “task dumping” if not accompanied by adequate supports and resources for staff to implement this new knowledge (Jacobs et al., 2021). Greater attention to role clarification, including aspects of MH/SU interventions that are within the remit of non-specialists, can help reduce scope creep and perceptions of task dumping. There are existing individual interventions and coping strategies for stress and burnout that researchers often include within task-shared MH trainings (Simms et al., 2023). Individual coping strategies for non-specialists to adopt may include spending quality time with friends and loved ones, religious practice and practicing self-care (De Hert, 2020). However, these individual strategies are not sufficient to address the scope of this issue; structural and systemic drivers of these stressors must also be addressed.

For multiple reasons, while they may reduce stigma, interventions to reduce non-specialist stress are unlikely to eliminate non-specialist stigma toward patients with MH/SU concerns, highlighting the need for MH/SU stigma reduction interventions targeting non-specialists in this setting. Initially, stigma reduction interventions in low- and middle-income countries focused on HIV-related stigma (Rao et al., 2019); however, trainings intended to reduce stigma toward MH/SU have also been developed and shown to be effective (Kaiser et al., 2022; Kohrt et al., 2022; Myers et al., 2024; Regenauer et al., in Press). These trainings tend to challenge stigmatizing attitudes and beliefs toward patients with SU/MH, addressing judgmental communication styles and providing the opportunity for non-specialists to have direct and indirect contact with people who have lived experience of MH and/or SU concerns.

Further, while patients of all identities must navigate patient-provider hierarchies within the healthcare system (Berndt and Bell, 2021; Scott et al., 2021), this can be particularly detrimental to patients such as those with HIV, MH and/or SU because of these already stigmatized identities (Ondenge et al., 2017; Kwame and Petrucka, 2020). Our findings also support the need for greater involvement of people with lived experience (PWLE) in anti-stigma program delivery and in healthcare more broadly (Thornicroft et al., 2022; World Health Organization, 2023). Their perspectives are critical for shaping provider behaviors in patient-centered and non-stigmatizing ways. PWLE should be involved in all levels of anti-stigma programs and placed in leadership roles, as recently recommended by the *Lancet Commission on Stigma and Discrimination* (Thornicroft et al., 2022). Including PWLE is a key component of program effectiveness, and having these individuals in leadership roles can help reduce self-stigma and may be impactful on their recovery journey (Thornicroft et al., 2022). Patient internalized and anticipated stigma must also be addressed alongside provider stigma, and research has shown that interventions targeting stigma may be effective for people living with HIV in various settings (Rao et al., 2012; Kalichman et al., 2019).

Findings highlight several areas of future research that have the potential to support more sustainable task-shared MH/SU programs and help non-specialist providers provide better quality care. Future task-shared MH/SU intervention studies should include outcomes focused on stress and stigma at multiple levels, while also working to identify necessary health systems interventions to address resource scarcity. Stigma interventions should include addressing enacted stigma from providers as well as internalized stigma experienced by patients. Inclusion of these outcomes simultaneously within studies would allow for a better understanding of how these constructs are interrelated and what implementation and intervention strategies can be used to address stress and stigma together within training and supervision programs for non-specialists taking on task-shared MH/SU care.

Findings should be interpreted in the context of study limitations. This study uses qualitative data collected to help inform the development of a stigma reduction intervention, so prompts included less direct probing on resource scarcity and stress, though both topics were organically brought up by participants and featured prominently throughout the interviews. The use of the inductive–deductive approach allowed authors to ensure that themes were driven by interviews instead of by a pre-set coding framework related only to the primary objective of the study (Fereday and Muir-Cochrane, 2006). Although not all participants spoke about these themes, the majority did, and further, frequency is not typically used as a metric for importance in qualitative research (Glaser, 2017). Many participants, particularly patients, did not actually use the term stigma. When one participant used the term “structural stigma”, there was no additional context that described this individual’s interpretation of this term, which is important to note. However, this participant was a program manager with a doctoral degree, suggesting that they may be familiar with the term. Our sample also includes perspectives from a relatively small sample of non-specialists and patients, and we do not have specific information on MH training providers may have had in the past. In the future, mixed methods research to explore resource scarcity, stress and stigma in larger samples could contribute to furthering the understanding of how these three phenomena interact.

## Conclusion

Resource constraints, non-specialist provider stress and stigma toward MH/SU conditions are common in health systems. As researchers continue to explore methods for supporting sustainable, high-quality task-shared MH/SU services within resource-limited settings, a better understanding of the constructs of stress and stigma and their interaction among non-specialist providers will be critical. Such an understanding can help identify coordinated interventions targeting both stress and stigma that can support non-specialists in undertaking task-shared MH/SU care and improve the quality of services. Further, a better understanding of how resource constraints drive stress and impact care quality can help shape advocacy for more resources for task-shared MH/SU care globally.

## Supporting information

Hines et al. supplementary material Hines et al. supplementary material

## Data Availability

Data are available upon reasonable written request to the corresponding author.

## Supplementary material

The supplementary material for this article can be found at http://doi.org/10.1017/gmh.2024.41.
