# Supplementary material for: “Early in the morning, there’s tolerance and later in the day it disappears” – The intersection of resource scarcity, stress and stigma in mental health and substance use care in South Africa
Source: Glob Ment Health (Camb). 2024 Apr 1;11:e45. doi: 10.1017/gmh.2024.41 (PMC11058524; doi:10.1017/gmh.2024.41)
Supplement: Hines et al. supplementary material [file S2054425124000414sup001.docx]

**Appendix A. Provider Interview Guide**

**Qualitative Interview Guide on CHWs and Stigma:**

**CHW, Provider and Stakeholder Version**

**Read aloud to participant:** This interview will take approximately 45 minutes. Please let me know if you need a break at any time. The reason for this interview is to better understand how you feel about working with patients who are struggling to stay engaged in TB and HIV services and your perspective on how we can better re-engage these patients. We want to understand your perspective on working with patients receiving TB and HIV care who are also struggling with mental health problems such as depression, alcohol, or drug use. Ultimately, we are trying to develop a new training programme for CHWs and your input will be very helpful to plan this programme.

With your permission, I will be audio-recording this session. Your name will not be on the recording. After the interview, the recording will be typed up into a transcript. If you happen to refer to a name during the session, the name will be removed from the transcript. What you share during this interview will in no way affect your role at the clinic or as a stakeholder in the healthcare system. Your responses in this interview will hopefully help us develop better care for people with HIV and TB who also have mental health problems, so we would appreciate if you can be as detailed and open as you can in your responses.

Any time you want to stop the interview or have me turn off the recording, you can tell me, and we will stop.

Do you have any questions before we start?

***(Turn on the recording)***

1. **Please describe your current work with patients with HIV and TB co-infection.**
2. **What are the main reasons why [your] patients receiving TB and HIV services fall out of care?**
   1. *Probe:* What about mental health issues, specifically depression or substance use (alcohol or drug use)?
3. **In your view, what are some of the challenges to retention in care for patients with mental health problems (such as depression, alcohol, or substance use)?**
   1. *Probe:* Why do you think they face these challenges?
   2. *Probe:* How can you best support patients with mental health problems to stay in TB/HIV care?
4. **What, if any, strategies have been helpful for re-engaging patients with TB and HIV co-infection back into care after they have disengaged?**
   1. *Probe*: Why do you think these have worked?
   2. *Probe:* What about strategies for patients who have depression or substance use problems?
5. **Please describe structural barriers to care, for example things like transportation costs or lack of housing, that could get in the way of reengaging patients with TB and HIV coinfection and mental health concerns back into care.**

*Try to probe depression, alcohol use, and drug use separately.*

***Questions for CHWs only***

1. *[Asked to CHWs]* **Please tell me what your experience is like working with patients with TB/HIV and mental health problems, such as depression, alcohol, or drug use.** [pause and elicit participant response]
2. *[Asked to CHWs]* **The next few questions ask about how you feel about working with different types of patients.**
   1. **How do you feel about working with patients with depression?** [elicit participant response]
      1. Describe how working with patients with depression affects your ability to engage them back into TB/HIV care.
   2. **How do you feel about working with patients with heavy? alcohol use?** [elicit participant response]
      1. Describe how working with patients with heavy alcohol use affects your ability to engage them back into TB/HIV care.
   3. **How do you feel about working with patients who use drugs?** [elicit participant response]
      1. Describe how working with patients who use drugs affects your ability to engage them back into TB/HIV care.
   4. **How do you feel about working with patients who are gay, lesbian, or transgender?** [elicit participant response]
      1. Describe how working with patients who have a different sexual orientation or gender identity, like being transgender, affects your ability to engage them back into TB/HIV care.
   5. **How do you feel working with patients living with HIV?** [elicit participant response] **How do you feel working with patients with TB?** [elicit participant response]
      1. Describe how working with patients with HIV and/or TB affects your ability to engage them back into TB/HIV care.

***Questions for stakeholders and other providers only- do not ask of CHWs***

1. *[Asked to stakeholders or other providers]* **Please describe your interactions to date with community health workers.** [elicit participant response].
   1. *Probe:* In what capacity do you currently (or have previously) work with CHWs? What were the extent of these interactions?.
2. *[Asked to stakeholders or other providers]* We are interested to understand how CHWs’ beliefs and attitudes may impact the care they provide, including stigma. By stigma, I mean negative beliefs about another person due to some personal characteristic. I want to hear your thoughts on how CHW stigma of different patient characteristics affects engaging patients in TB/HIV care.
   1. **How common is CHW stigma towards patients with depression?** [elicit participant response] **How does this attitude affect CHWs’ work reengaging patients in TB/HIV care?**
   2. **How common is CHW stigma toward patients with substance use problems, either alcohol or drug use?** [elicit participant response] **How does this attitude affect CHWs’ work reengaging patients in TB/HIV care?**
   3. **How common is CHW stigma towards patients who are gay, lesbian, or transgender?** [elicit participant response] **How does this attitude affect CHWs’ work reengaging patients in TB/HIV care?**
   4. **How common is CHW stigma due to the fact that patients are living with HIV and/or TB?** [elicit participant response] **How does this attitude affect CHWs’ work reengaging patients in TB/HIV care?**

***Questions for all providers***

1. **We would like to develop a training to help CHWs more effectively re-engage patients with TB/HIV who have depression and substance use back into care.**
   1. What are some strategies that you think would help CHWs improve their work with patients with depression and/or substance use?
   2. Are there any cultural factors such as local customs, beliefs, and/or practices related to depression and/or substance use that would support such a training programme or be a barrier?
2. **What might get in the way or be a barrier to implementing a training programme to reduce CHW’s negative attitudes or stigma towards patients with depression and substance use?**
3. **What might help or facilitate implementing a training programme to reduce CHW’s stigma towards patients with depression and substance use?**
4. **Please describe examples of people you know in your life that have recovered from mental health or substance use problems.** [elicit participant response]
   1. Describe how your exposure to this person(s) has changed your view of mental health or substance use problems, if at all. Why or why not?
5. **How would you feel about someone with lived experience, for instance with mental health or substance use problems, joining a training for CHWs to share their story?** [elicit participant response]

*Probes:*

- 1. In what ways would this be helpful?
  2. In what ways would this be harmful?
  3. What do you think about this as an approach to shifting attitudes and beliefs?
  4. How would you change this approach? Why?

1. **We want to design a training program that would be feasible and realistic for CHWs to attend.**
   1. Please describe what is feasible for you (or other CHWs) to attend for training at this time (i.e., length, timing).
   2. How feasible would it be for CHWs to attend weekly supervision of one hour to discuss clinical issues? Please explain your response.
2. **What additional training needs do you (or CHWs in general) have that you think would improve engagement in HIV/TB care for people with mental health and/or substance use problems?**

**Please let us know if there are additional questions or topics that we did not cover today that you think are relevant. We would like to hear any additional thoughts or considerations you have.**

**Your input and expertise on these topics is incredibly valued as we design a new CHW training program based on this feedback. Thank you again for your time and participation.**

**Appendix B. Patient Interview Guide**

**Qualitative Interview Guide on CHWs and Stigma:**

**Patient Version**

**Read: This interview will take approximately 45 minutes. Please let me know if you need a break at any time. The reason for this interview is to better understand how you feel about working with CHWs who have tried to support you to stay in your TB/HIV care. We want to understand your perspective on working with these providers, specifically what’s been helpful and unhelpful. Ultimately, we are trying to develop a program to better train CHWs in working more effectively with patients like yourself. Your input will be very helpful to plan this program.**

**With your permission, I will be audio-recording this session. Your name will not be on the recording. After the interview, the recording will be typed up into a transcript. If you happen to refer to a name during the session, the name will be removed from the transcript. What you share during this interview will in no way affect the services or treatment you receive at the clinic you attend or in any other healthcare setting. Your responses in this interview will hopefully help us develop better care to support people with HIV and TB to stay engaged in care, so we would appreciate if you can be as honest as you can in your responses.**

**Any time you want to stop the interview or have me turn off the recording, you can tell me, and we will stop. Do you have any questions before we start?**

***(Turn on the recording)***

1. **Please can you tell me about your HIV and TB treatment experiences.**
2. **What have been the main challenges you have had with staying in care for HIV and TB treatment?**
   1. ***Probe:*** What about mental health issues, specifically depression or substance use (alcohol or drug use)?
   2. Were some of these challenges specifically related to TB or HIV treatment (i.e., *probe TB vs. HIV separately).*
   3. ***Other areas to probe:*** Transportation, lack of stable housing, treatment costs
3. **Please tell me about your experiences working with CHWs who have come to your home.**
   1. ***Probe:*** How often (or when) have you met with a CHW in your home?
   2. ***Probe:*** Why did they visit?
   3. ***Probe:*** What is your understanding of the role of the CHW?
4. **What were your interactions like with the CHW(s)?**
   1. ***Probe:*** Tell me about any experiences where you felt supported by the CHW when they tried to get you back into care. What did he/she do to make you feel this way?
   2. ***Probe:*** Tell me about any experiences you have had where you felt judged or blamed by the CHW when they tried to get you back into care. What did he/she do to make you feel this way?
5. **We want to hear how you think CHWs could help patients like you stay in care.**
   1. ***Probe (Information):*** What information would be helpful, or has been helpful?
   2. ***Probe (Motivation):*** What would motivate you, or has motivated you?
   3. ***Probe (Behavior):*** What behaviors do you need support with to stay engaged in care (*or what behaviors did you need support with in the past?*)?
6. **What else do you think would help TB/HIV patients who have fallen out of care get back into care? Your ideas don’t necessarily have to be about CHWs.**
7. **We are interested to understand how CHWs’ beliefs and attitudes affect the care they provide, including stigma. By stigma, I mean negative beliefs about another person due to some personal characteristic. I want to hear your thoughts on CHWs’ beliefs towards different patient characteristics, and how these beliefs, or stigma, may affect patients in TB/HIV care.**
   1. **CHW stigma due to the fact that patients are living with HIV and/or TB?** [elicit participant response]
      1. **In your view, how does this CHW stigma affect their work to help patients get back to TB/HIV care?**
      2. **Have you had any personal experiences of these attitudes [or stigma] from CHWs?**
         1. ***Probe:*** If so, how has it affected you? How has it affected your TB/HIV care?
   2. **CHW stigma towards patients with depression?** [elicit participant response]
      1. **In your view, how does this CHW stigma affect their work to help patients get back to TB/HIV care?**
      2. **Have you had any personal experiences of these attitudes [or stigma] from CHWs?**
         1. ***Probe:*** If so, how has it affected you? How has it affected your TB/HIV care?
   3. **CHW stigma toward patients with substance use problems, either alcohol or drug use?** [elicit participant response]
      1. **In your view, how does this CHW stigma affect their work to help patients get back to TB/HIV care?**
      2. **Have you had any personal experiences of these attitudes [or stigma] from CHWs?**
         1. ***Probe:*** If so, how has it affected you? How has it affected your TB/HIV care?
   4. **CHW stigma towards patients who are gay, lesbian, or transgender?** [elicit participant response]
      1. **In your view, how does this CHW stigma affect their work to help patients get back to TB/HIV care?**
      2. **Have you had any personal experiences of these attitudes [or stigma] from CHWs?**
         1. ***Probe:*** If so, how has it affected you? How has it affected your TB/HIV care?
   5. **CHW stigma towards patients who are Black or Coloured?** [elicit participant response]
      1. **In your view, how does this CHW stigma affect their work to help patients get back to TB/HIV care?**
      2. **Have you had any personal experiences of these attitudes [or stigma] from CHWs?**
         1. ***Probe:*** If so, how has it affected you? How has it affected your TB/HIV care?
8. **We would like to develop a training to help CHWs work better with patients with TB/HIV who also have depression and substance use. What do you think CHWs need to know about depression and substance use?**
   1. ***Probe:*** What would be most helpful for them to know about depression and substance use to help you stay engaged in care?
   2. ***Probe: In what ways does your culture, tradition, or beliefs influence your understanding of*** depression and substance use and how it can be treated ?
9. **How would you feel about working with a provider, such as a CHW, with lived experience, or a common identity with you? This “common identity” means the person would be similar to you and may have struggled with some of the same health issues as you.** [elicit participant response]
   1. ***Probe:*** What aspect of your identity would you most want this person to have?
   2. ***Probe:*** Would you prefer this person to be living with HIV? TB? Why?
   3. ***Probe:*** Would you prefer this person to have depression? Why?
   4. ***Probe:*** Would you prefer this person to have alcohol use? Why?
   5. ***Probe:*** Would you prefer this person to have drug use? Why?
   6. ***Probe:*** Would you prefer this person to be experiencing or in recovery from these issues [depression, substance use]. Why?
10. **How would you feel about sharing your own story with other CHWs as part of their training?**
    1. ***Probe:*** What would be your main concerns?

*Please let us know if there are additional questions or topics that we did not cover today that you think are relevant. We would like to hear any additional thoughts or considerations you have.*

*Your input and expertise on these topics is incredibly valued as we design a new CHW training program based on this feedback. Thank you again for your time and participation.*
