# Supplementary material for: “Early in the morning, there’s tolerance and later in the day it disappears” – The intersection of resource scarcity, stress and stigma in mental health and substance use care in South Africa
Source: Glob Ment Health (Camb). 2024 Apr 1;11:e45. doi: 10.1017/gmh.2024.41 (PMC11058524; doi:10.1017/gmh.2024.41)
Supplement: Peer review history

# Editor report: Recommendation: “*Early in the morning, there’s tolerance and later in the day it disappears*” – The intersection of resource scarcity, stress and stigma in mental health and substance use care in South Africa — R0/PR2

**DOI:** 10.1017/gmh.2024.41.pr2

**Affiliations:** The George Institute for Global Health India, India

Please address the comments made by the reviewers. In particular i feel the theme 3 is critical and needs more information. the quotes provided are quite superficial to justify the theme. Also one is not sure how structural stigma is being understood by the participants as it is a term that even many researchers are not clear about. So it was surprising that ‘structural stigma’ was being quoted.

# Editor report: Decision: “*Early in the morning, there’s tolerance and later in the day it disappears*” – The intersection of resource scarcity, stress and stigma in mental health and substance use care in South Africa — R0/PR3

**DOI:** 10.1017/gmh.2024.41.pr3

**Affiliations:** Johns Hopkins University Bloomberg School of Public Health, United States

No accompanying comment.

# Editor report: Recommendation: “*Early in the morning, there’s tolerance and later in the day it disappears*” – The intersection of resource scarcity, stress and stigma in mental health and substance use care in South Africa — R1/PR5

**DOI:** 10.1017/gmh.2024.41.pr5

**Affiliations:** The George Institute for Global Health India, India

Pl address the comments by one reviewer. 

I can also suggest checking the Lancet Commission on Stigma and discrimination from 2023 which does allude to the importance of involving PWLE and has specific sections and recommendations which may be relevant to your paper.

# Editor report: Decision: “*Early in the morning, there’s tolerance and later in the day it disappears*” – The intersection of resource scarcity, stress and stigma in mental health and substance use care in South Africa — R1/PR6

**DOI:** 10.1017/gmh.2024.41.pr6

**Affiliations:** Johns Hopkins University Bloomberg School of Public Health, United States

No accompanying comment.

# Editor report: Recommendation: “*Early in the morning, there’s tolerance and later in the day it disappears*” – The intersection of resource scarcity, stress and stigma in mental health and substance use care in South Africa — R2/PR8

**DOI:** 10.1017/gmh.2024.41.pr8

**Affiliations:** The George Institute for Global Health India, India

No accompanying comment.

# Editor report: Decision: “*Early in the morning, there’s tolerance and later in the day it disappears*” – The intersection of resource scarcity, stress and stigma in mental health and substance use care in South Africa — R2/PR9

**DOI:** 10.1017/gmh.2024.41.pr9

**Affiliations:** Johns Hopkins University Bloomberg School of Public Health, United States

No accompanying comment.
